# Health Monitoring of Large-Scale Civil Structures: An Approach Based on Data Partitioning and Classical Multidimensional Scaling

**DOI:** 10.3390/s21051646

**Published:** 2021-02-26

**Authors:** Alireza Entezami, Hassan Sarmadi, Behshid Behkamal, Stefano Mariani

**Affiliations:** 1 Department of Civil and Environmental Engineering, Politecnico di Milano, Piazza L. da Vinci 32, 20133 Milano, Italy; stefano.mariani@polimi.it; 2 Department of Civil Engineering, Faculty of Engineering, Ferdowsi University of Mashhad, Mashhad 9177948944, Iran; hassan.sarmadi@mail.um.ac.ir; 3 Department of Computer Engineering, Faculty of Engineering, Ferdowsi University of Mashhad, Mashhad 9177948944, Iran; behkamal@um.ac.ir

**Keywords:** structural health monitoring, data-driven method, high-dimensional data, classical multidimensional scaling

## Abstract

A major challenge in structural health monitoring (SHM) is the efficient handling of big data, namely of high-dimensional datasets, when damage detection under environmental variability is being assessed. To address this issue, a novel data-driven approach to early damage detection is proposed here. The approach is based on an efficient partitioning of the dataset, gathering the sensor recordings, and on classical multidimensional scaling (CMDS). The partitioning procedure aims at moving towards a low-dimensional feature space; the CMDS algorithm is instead exploited to set the coordinates in the mentioned low-dimensional space, and define damage indices through norms of the said coordinates. The proposed approach is shown to efficiently and robustly address the challenges linked to high-dimensional datasets and environmental variability. Results related to two large-scale test cases are reported: the ASCE structure, and the Z24 bridge. A high sensitivity to damage and a limited (if any) number of false alarms and false detections are reported, testifying the efficacy of the proposed data-driven approach.

## 1. Introduction

Civil structures such as buildings, bridges, towers, subways, tunnels, dams are valuable structural systems that play a crucial role in social life and transportation networks. Damage and deterioration/aging processes are serious hazards to the safety and health of these structures. In recent times, structural health monitoring (SHM) has become a key tool to assess the integrity and health of such structures. Vibration-based SHM methodologies are reliable approaches to monitor the dynamic characteristics of the systems and detect structural damage [1,2,3]. Generally speaking, there are two possible approaches to the SHM problem: model-based, and data-driven methods.

A model-based method needs an accurate numerical (e.g., finite element) model of the real structure in order to define the physical properties and/or modal parameters as information regarding the relevant virgin or undamaged state. By exploiting the experimentally measured dynamic characteristics obtained via a sensor network deployed over the structure, it is possible to detect, locate and quantify possible damage patterns by means of model updating procedures [4,5]. These procedures attempt to reduce the discrepancy between the outputs of the numerical model and the real-life data [6]. One major disadvantage of these methods is that model updating is typically necessary even before attacking the damage detection problem, to allow for epistemic uncertainties. Furthermore, this makes it difficult, if not impossible, to collect information linked to a large number of mode shapes. Hence, model reduction techniques appear to be necessary.

In contrast, a data-driven method handles raw vibration measurements without any of the limitations that are typical of the model-driven approach. Data-driven methods can be implemented either in an offline or online fashion, see [7,8]. Most of the data-driven methods are based on statistical pattern recognition [9,10,11]. The main idea behind this is to extract damage-sensitive features from the vibration measurements acquired through various types of sensors, such as, e.g., optical fibers [12,13], piezoelectric transducers [14], or MEMS [15]. The design of optimal sensor placement has to be performed, starting with data acquisition [16,17].

Time series analysis is a powerful method for modeling the measured structural vibrations and extracting the mentioned damage-sensitive features [18]. In this regard, the AutoRegressive (AR) time series representation is one of the most effective methods. Due to major benefits, such as simplicity, sensitivity to damage, compliance with output-only setups without any requirement of input data (in terms of, e.g., excitation forces), and independence of the excitation source, AR modeling is widely used in data-driven SHM applications [19,20,21,22].

Once damage-sensitive features have been extracted from the dataset, the final step of a data-driven SHM method is to analyze the features themselves for decision-making, providing outcomes in terms of early damage detection, localization, and quantification. At this stage, different techniques can be adopted, including statistical distance metrics (e.g., the Mahalanobis distance [23,24] or the Kullback–Leibler divergence [10,21,25]), Bayesian approaches [26,27], artificial neural networks [28,29], principal component analysis [30,31], and clustering [32,33,34]. In spite of their applicability, they may not perform efficiently when damage-sensitive features are of a high-dimensional nature, namely in the presence of big data to process; this leads to a time-consuming and unreliable decision-making process [10,35]. Although reductions in the size of datasets is the standard choice to address the big data issue, a major concern is related to the fact that important information regarding the structural health may be lost due to data reduction.

Another challenging issue within the context of SHM is provided by environmental variations (e.g., in terms of fluctuations of temperature, wind speed, humidity, etc.), that can give rise to subtle changes in the vibration responses that mask those actually caused by damage. As the features extracted from the structural response can also be sensitive to environmental variability, damage detection may suffer from false alarms and erroneous damage detection results [36]. Even though research activities have already investigated how to cope with the effects of such environmental variability, this is still an open challenge, especially when the extracted features are high-dimensional ones.

Accordingly, the main objective of the present study is to propose a novel data-driven method for damage detection, addressing the limitations linked to high-dimensional data, with the ability to process data without any concerns regarding the loss of information about the structural state. This method is based on a simple, yet effective data partitioning strategy and on classical multidimensional scaling (CMDS), which gives this method the ability to work in low-dimensional feature spaces and define proper indices for damage detection. To the best of the authors’ knowledge, here, the CMDS algorithm is used for the first time in the context of damage detection. A great advantage of the proposed method is the efficiency in dealing with high-dimensional data under strong environmental variability, while also providing reliable and accurate damage detection outputs. These conclusions are arrived at by assessing the performance of the approach against two well-known, full-scale benchmark examples: the American Society of Civil Engineers (ASCE) structure, and the Z24 Bridge.

The remainder of the paper is organized as follows. In Section 2, the CMDS procedure is discussed in order to obtain insights into the properties of the algorithmic coordinates characterizing the structural state. Section 3 provides details regarding the subdivision of the high-dimensional feature samples and the computation of the damage indices. In Section 4, outcomes are discussed for the two aforementioned benchmark real-life cases, pointing out the accuracy and reliability of damage estimation. Finally, Section 5 gathers some concluding remarks on the proposed approach, and provides suggestions for future research to further improve the proposed SHM scheme.

## 2. Classical Multidimensional Scaling

Multidimensional scaling (MDS) is a statistical data analytics strategy that quantifies the (dis)similarity between datasets through distances in a low-dimensional space, to make them accessible for visual inspection and exploration. For observations featuring different correlation levels, the MDS representation is given for the plane that collects samples with the highest values of the correlation itself [37]. MDS hence provides a graphical visualization that enables the inspection of the structure in the dataset.

In CMDS, it is assumed that the dissimilarities between datasets are provided as pairwise distances between pairs of objects; relevant coordinates to measure these distances are searched for. The CMDS algorithm, thus, takes the distance matrix **D***_n_*_×*n*_ from a multivariate dataset **X***_n_*_×*r*_ as the input, and returns a coordinate matrix **U***_n_*_×*q*_ (with *q* < *n*) [37]. Here, *q* is automatically set by finding the smallest space in which the inter-point distances collected in **D***_n_*_×*n*_, which are therefore relevant to all the *n* points, are zero [38]. For this purpose, the Euclidean-squared distance (ESD) is adopted to measure pairwise distances among all the data points. According to this scheme, pairwise ESDs are given for all the row vectors of the matrix **X***_n_*_×*r*_, and are then collected in the matrix **D***_n_*_×*n*_.

Given the multivariate dataset **X***_n_*_×*r*_, which is made of *n* row vectors **x**_1_, **x**_2_, ..., **x***_n_* of *r* variables, the ESD between **x***_j_* and **x***_k_* is defined as follows:

(1) $$d_{E}^{2}(j,k)=\left(x_{j}-x_{k}\right){\left(x_{j}-x_{k}\right)}^{T}.$$

where *j*, *k* = 1, …, *n*. In this way, the marginal means are removed from the multivariate dataset. By computing the distances for all the possible vector pairs in **X**, the distance matrix **D** is obtained as:

(2) $$D_{n\times n}=\left[\begin{array}{ccccc} 0 & d_{E}^{2}(1,2) & d_{E}^{2}(1,3) & \cdots & d_{E}^{2}(1,n)\\ d_{E}^{2}(2,1) & 0 & d_{E}^{2}(2,3) & \cdots & d_{E}^{2}(2,n)\\ \vdots & \vdots & \vdots & \vdots & \vdots \\ d_{E}^{2}(n,1) & d_{E}^{2}(n,2) & d_{E}^{2}(n,3) & \cdots & 0 \end{array}\right].$$

The distance matrix **D** is next transformed into the matrix **B**, by applying a double-centering operation according to:

(3) $$B_{n\times n}^{}=-\frac{1}{2}J_{n\times n}^{}D_{n\times n}^{}J_{n\times n}^{}$$

where the centering matrix **J***_n_*_×*n*_ is defined as:

(4) $$J_{n\times n}=I_{n\times n}-\frac{1}{n}1_{n\times 1}{\left(1_{n\times 1}\right)}^{T}.$$

In Equation (4), **I***_n_*_×*n*_ denotes an *n*-by-*n* identity matrix and **1***_n_*_×1_ is an *n*-dimensional vector with unitary entries.

The MDS coordinates are then obtained from **B** by means of the following eigen-decomposition:

(5) $$B_{n\times n}=Q_{n\times n}\Lambda_{n\times n}Q_{n\times n}^{T}$$

where **Q** and **Λ** are the orthogonal eigenvector and diagonal eigenvalue matrices, respectively. Let the first *q* eigenvalues be collected in **Λ***_n_*_×*q*_, and the corresponding eigenvectors collected in **Q***_n_*_×*n*_; the coordinate matrix **U** of CMDS is finally given by:

(6) Un×q=Qn×nΛn×q12.

Since the coordinate matrix **U** is obtained from the distance matrix **D**, whose entries have been provided by the ESD, in order to set *q*, it is necessary to find the smallest space with zero inter-point distances, without the adoption of any additional technique. By means of Equation (1), as the distance of a vector from itself is always zero, moving to all the *r* vectors of matrix **X**, one can automatically figure out that *q* = *r*.

It is worthy of note, that the CMDS algorithm is based on the eigen-decomposition of Equation (5), which means that it may resemble a principal component analysis (PCA). However, the two algorithms actually differ, as detailed in the following. In general, the input to the PCA is provided by the original data samples; the PCA algorithm handles these data and projects them onto the directions characterized by the highest variance. As discussed above, the input to CMDS is instead represented by the pairwise distances among the original data samples; this allows one to design a distance network. The output of CMDS is represented by projections of the samples onto reduced-order planes or spaces, wherein distances are preserved [39]. Further to this, it is worth stressing that PCA can be considered as a parametric algorithm, and the number of principle components to be retained in a reduced order model is its main hyperparameter to be set. Here, by hyperparameter we mean any unknown component/parameter of the algorithm that affects the model performance that plays a prominent role and therefore must be set properly. In contrast, CMDS is a non-parametric algorithm, which means that it does not rely upon unknown parameters to be set at the implementation level. This conclusion stems from the fact that the only parameter to set is *q*, which is automatically determined by the procedure. Accordingly, CMDS is expected to prove to be more efficient than PCA for the current purposes.

## 3. Proposed SHM Data-Driven Method via CMDS

Early damage detection, representing the first stage of damage diagnosis, is mainly intended to ascertain whether a damage pattern has been triggered anywhere in the structural system. This stage of SHM aims at assessing the global state of the structure, so as to distinguish any damaged condition from the normal, healthy or virgin state. Although this process is simpler than those related to the other levels of damage diagnosis (namely damage localization and quantification), it may turn out to be difficult to carry out if the features extracted from the structural vibrations are high-dimensional ones and if environmental variability causes deceptive changes in them, in a way similar to damage. The proposed data-driven method initially exploits a simple but effective partitioning strategy, leading to low-dimensional feature spaces and effective outputs for damage detection, to then feed the CMDS algorithm for damage detection.

Let **X***_n_*_×*r*_ and **Z***_m_*_×*r*_ be the sets of damage-sensitive features related to the healthy and current states of the structure, respectively. In these sets, *n* and *m* are the numbers of feature samples (e.g., AR residuals or modal frequencies), and *r* denotes the number of variables (e.g., the number of sensors deployed over the structure) related to the state of interest. As the matrices include high-dimensional feature samples, in other words as *n* ≫ *r* and *m* ≫ *r*, the CMDS algorithm yields large matrices **D**, **J**, **B** and **U**. The direct use of this algorithm may, thus, lead to a time-consuming and computationally inefficient strategy for SHM.

**Figure 1:**
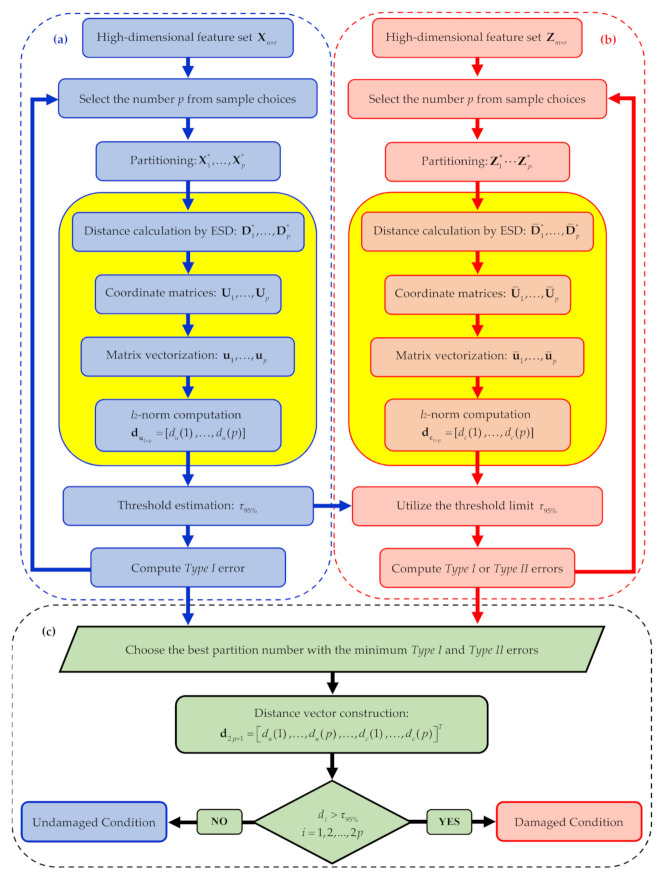
Graphical flowchart of the proposed classical multidimensional scaling (CMDS)-based structural health monitoring (SHM) method: (**a**) iterative loop related to the undamaged condition, (**b**) iterative loop related to the current state, (**c**) decision-making strategy based on the chosen number *p* of partitions. The yellow boxes are specifically related to the CMDS algorithm.

To deal with the feature sample size problem, low-dimensional sets of the same features must be obtained by partitioning the matrices **X** and **Z** into *p* sub-sets $X_{1}^{*}\ldots X_{p}^{*}$ and $Z_{1}^{*}\ldots Z_{p}^{*}$, featuring a (far) smaller number of samples. To set *p*, the criterion here adopted is based on the SHM method performance in terms of Type I and Type II errors. To this purpose, a set of values of *p* are defined and the error rates are computed for the undamaged and also the current states; finally, the value leading to the smallest amount of errors is selected. This iterative procedure is described in the flowchart in Figure 1. It is noteworthy that, in this procedure, the distance values for decision-making are computed only once the optimal value of *p* has been obtained, see stage (c). Accordingly, the risks related to false alarm (Type I) and false detection (Type II) errors are automatically minimized. In the following, all the steps of the procedure are described in detail.

Regarding the two iterative loops (a) and (b) for the undamaged and current states to be compared, the available feature matrices **X** and **Z** are partitioned into two sets of smaller ones of sizes *l* × *r* and *h* × *r*, respectively, where *l* ≪ *n* and *h* ≪ *m*. With such low-dimensional feature sets to handle, the CMDS algorithm is adopted to determine the relevant distance matrices: for all these partitions, the distance matrices turn out to be of sizes *l* × *l* and *h* × *h*, respectively. With the CMDS algorithm, the *l* × *r* coordinate matrices **U**_1_, …, **U***_p_* for $X_{1}^{*},\ldots ,\;X_{p}^{*}$ and the *h* × *r* coordinate matrices **Ū**_1_,…, **Ū***_p_* for $Z_{1}^{*},\ldots ,Z_{p}^{*}$ are obtained through Equations (3)–(6).

The matrix vectorization technique is next employed to convert the coordinate matrices into vectors **u**_1_…**u***_p_* and **ū**_1_, …, **ū***_p_*, respectively, featuring l* and h* samples, where l* = *l* × *r* and h* = *h* × *r.* The sought indices for damage detection are finally given by the *l*_2_-norms of these vectors, according to:

(7) $$d_{u}(i)=\sqrt{\sum_{k=1}^{l^{*}}{\left|u_{i}\left(k\right)\right|}^{2}}$$

(8) $$d_{c}(i)=\sqrt{\sum_{k=1}^{h^{*}}{\left|{\bar{u}}_{i}\left(k\right)\right|}^{2}}$$

where *I* = 1, …, *p,* and subscripts *u* and *c*, respectively, denote the undamaged and current states of the structure. The norm values are all collected in a damage indicator vector **d**, defined in the following form:

(9) $$d_{2p\times 1}={\left[\begin{array}{cc} d_{u} & d_{c} \end{array}\right]}^{T}.$$

where **d_u_** = [$d_{u}\left(1\right)$, …, $d_{u}\left(p\right)$] and **d_c_** = [$d_{c}\left(1\right)$,… , $d_{c}\left(p\right)$] are the corresponding *p*-dimensional vectors related to all the partitions in the two states to be compared.

By means of vectors $d_{u}\;$ and $d_{c}$, along with a threshold limit, it is possible to ascertain the effects of *p* on the number of Type I and Type II errors in the iterative loops of the procedure. Even if damage indices are computed and compared with the threshold limit during these stages, nothing is done regarding the final decision-making to assess the current structural state. Regarding loop (a), since data and features refer to the undamaged condition, only Type I errors need to be checked. In loop (b) instead, for any new information collected and related to the current and unknown state, the distance values in **d_c_** are computed and compared with the threshold limit set with the undamaged state. Since this iterative loop is intended to optimize the dataset partitioning, both Type I and Type II errors are assessed by means of the results attained in loop (a); this is necessary since, as already remarked, the current state is unknown and can be either undamaged or damaged.

In what precedes, the threshold is set on the basis of the entries in vector **d_u_** and regarding the undamaged state. If these values are assumed to be normally or nearly normally distributed, the standard confidence interval based on a significance level can be used to define the threshold. For instance, by a 5% significance level, an upper bound on the 95% confidence interval for the *l*_2_-norms of the vector **d_u_** provides the threshold value as:

(10) $$\tau_{95\%}=\mu_{u}+1.96\;\sigma_{u}$$

where *μ_u_* and *σ_u_* are the mean value and standard deviation of the entries of **d_u_**. Accordingly, the *l*_2_-norm values of **d_u_** are all expected to fall below the threshold limit, while if the structure has undergone any damage the *l*_2_-norm values in vector **d_c_** are supposed to exceed the same threshold, to warn about damage occurrence.

Once the optimal value of *p* has been selected by minimizing the classification errors, decision-making for early damage detection is carried out on the current state by comparing the entries in **d_c_** with the threshold limit, as reported in stage (c) of Figure 1. In compliance with the proposed procedure, in the following results section, the charts regarding the damage indicator vector will provide the first *p* values related to the normal condition, all to fall below the threshold limit stating that the structure is undamaged. In case of damage occurrence, the next *p* values relevant to the current state will show a drift away from those corresponding to the normal condition, and exceed the threshold limit if classification errors do not show up.

## 4. Experimental Validation

### 4.1. ASCE Structure—Phase II

The effectiveness of the proposed method is first assessed against the experimental datasets relevant to the four-story steel structure of the second phase of the ASCE problem [40]. This structure consists of a 2-bay-by-2-bay steel frame, which is 2.5 × 2.5 m in plan and 3.6 m tall. The members were made of hot-rolled 300 W grade steel, with a nominal yield stress of 300 MPa. In each bay, the bracing system was represented by two threaded steel rods with a diameter of 12.7 mm, placed in parallel along the diagonal.

To obtain a realistic mass distribution, four 1000 kg slabs were placed on the first, second, and third floors, while four 750 kg slabs were placed on the fourth one. On each floor, two of the masses were placed off-center to increase the degree of coupling between the translational motions of the structure. The structure was subjected to a random excitation via an electro-dynamic shaker, placed on the fourth floor. The vibration responses, in terms of acceleration time histories, were acquired with 15 accelerometers (with three of them for each story) and a frequency of data acquisition of 250 Hz. Table 1 provides the sensor numbering and their locations for this benchmark problem: Sensors #1–3 are not listed in it and are not considered here, since they were mounted on the basement of the structure and did not provide relevant information concerning its dynamics.

During the tests, the damage was simulated by removing several braces from the east, southeast, and north sides of the structure, or by loosening bolts at the beam–column connections. In this work, only the damage patterns caused by removing the bracing systems from the east and southeast sides have been considered, see Table 2. Response modeling and feature extraction were first performed by time series analysis, via an AR model. The AR residuals at all sensor locations, regarding both the undamaged and damaged states, were then adopted as damage-sensitive features. Additional details about the residual-based feature extraction algorithm based on AR modeling and other time series analyses, can be found in [19].

The Leybourne–McCabe (LMC) hypothesis test was then adopted to ascertain the stationarity of the vibration signals and also the compatibility with the proposed modeling strategy [41]. As outputs, the LMC test provides the null (H_0_) or alternative (H_1_) hypothesis, a probability value (*p*-value), a critical value (*c*-value) and a test statistic (*Q*). The AR model is suitable for a univariate time series if the *p*-value is larger than a significance limit (*α*), or if the test statistic is smaller than the *c*-value. For example, under a customarily adopted 5% significance level (namely, for *α* = 0.05), *p*-value > 0.05 and/or *Q* < *c*-value = 0.1460 the mentioned stationarity of the time series is ascertained. Table 3 lists the LMC test statistics for all the sensor locations of Cases 1–5, based on such 5% significance level: since all the values of *Q* are smaller than the *c*-value = 0.1460, the measured vibration responses can be considered stationary in all the cases, and conform to an AR model. This model thus appears to be accurate for the current feature extraction purposes.

Next, the AR model order has to be set. The determination of an optimal order is of paramount importance for the process of feature extraction via time series modeling, in order to avoid issues related to a poor goodness-of-fit. In this work, the approach proposed in [19] has been adopted, to obtain the mentioned model order at each sensor location and for the undamaged Case 1. This algorithm is based on the residual analysis by the Ljung–Box hypothesis test, to assess the correlation between the model residuals. As an appropriate model order should enable the time series representation to generate uncorrelated residuals, their uncorrelatedness was selected as the criterion to set the model order [19].

If the residual sequences of the time series model are uncorrelated, the *p*-value provided by the Ljung–Box Q (LBQ) test becomes larger than the significant limit, while the test statistic remains smaller than the *c*-value. The smallest model order to satisfy these selection criteria was chosen as the order to use for feature selection purposes. Table 4 displays the model orders for sensors #4–15 and Case 1, as well as the obtained *p*-values: all the *p*-values are shown to be larger than the 5% significance limit, with the H_0_ hypothesis satisfied at all sensor locations. By adopting such model orders, the coefficients of all the AR models for the undamaged state have been then estimated by the least-squares technique. Finally, the model residuals for Cases 1–5 have been extracted and handled as the damage-sensitive features by means of the residual-based extraction technique described in [19].

In the next step, the residual samples at all the sensor locations for Case 1 (undamaged state) and Cases 2–5 (current states) have been collected into two different sets, to provide the feature matrices **X** and **Z**, see Section 3. These matrices collect *n = m* = 24,000 samples (as rows) and *r* = 12 variables (as columns). Before classifying Cases 2–5 as damaged states or not, it is necessary to set the optimal number *p* of partitions: the ten sample values reported in Table 5 have been investigated. In the table, results are reported for each of them in terms of number and percentage of Type II errors for Cases 4–5 (Table 5); results are instead not reported for Cases 2–3, since no errors at all were encountered, independently of *p*. As all the distance values relevant to Case 1 have been shown to fall below the threshold limit, without Type I errors, the optimal value of *p* has been set accordingly on the basis of the rates of Type II errors only. Focusing on Cases 4 and 5, values *p* ≤ 20 are shown to yield the best performances: hence, *p* = 20 has been adopted in the current analysis. The feature matrices X and Z were then subdivided into 20 sub-sets $X_{1}^{*}\ldots X_{20}^{*}$ and $\;Z_{1}^{*}\ldots Z_{20}^{*}$, each of which consisting of 1200 samples (*l* = *h* = 1200) and, again, 12 variables. The 1200-by-1200 distance matrices $D_{1}^{*}\ldots D_{20}^{*}$ for $\;X_{1}^{*}\ldots X_{20}^{*}$, and ${\bar{D}}_{1}^{*}\ldots {\bar{D}}_{20}^{*}$ for $\;Z_{1}^{*}\ldots Z_{20}^{*}$ were computed using the ESD technique.

**Figure 2:**
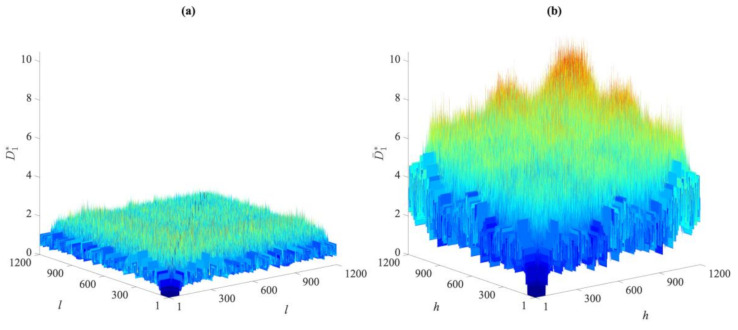
ASCE problem: comparison between the distance values in matrices (**a**) $D_{1}^{*}$ for Case 1, and (**b**) ${\bar{D}}_{1}^{*}$ for Case 2.

**Figure 3:**
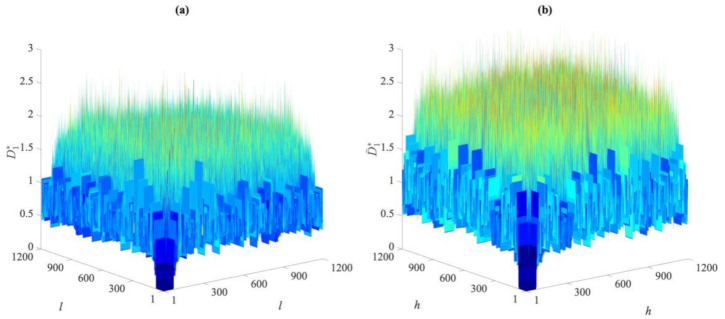
ASCE problem: comparison between the distance values in matrices (**a**) $D_{1}^{*}$ for Case 1, and (**b**) ${\bar{D}}_{1}^{*}$ for Case 5.

To visually compare the entries of these distance matrices relevant to the undamaged and damaged states, Figure 2 and Figure 3 show the exemplary cases of the first partition in Cases 1, 2, and 5. As reported in Figure 2, there are clear differences between the distance values gathered by the two matrices for Cases 1 and 2. The damage in the second case led to remarkable increases in the distance values in the matrix ${\bar{D}}_{1}^{*}$. On the contrary, by comparing the entries of $D_{1}^{*}$ and ${\bar{D}}_{1}^{*}$, respectively, related to Cases 1 and 5 and shown in Figure 3, it is difficult, if not impossible, to ascertain the presence of damage in the latter one due to the very similar values reported. The results have been shown for distances in the first partitions, collected in $D_{1}^{*}$ and ${\bar{D}}_{1}^{*}$, but they were very similar when also using the others. This brings us to the conclusion that the direct comparison of the distance values may be neither efficient nor informative, and the proposed data-driven approach for early damage detection therefore appears to be necessary.

Once the distance matrices for all the partitions regarding both the undamaged and the damaged conditions have been determined, the coordinate matrices **U**_1_…**U**_20_ and **Ū**_1_…**Ū**_20_ can be computed according to Equations (3)–(6), each one gathering 1200 samples and 12 variables, where *q* = *r* = 12 has been automatically set without any hyperparameter optimization tool, as explained in Section 2. The matrix vectorization technique was adopted next to obtain the vectors **u**_1_…**u**_20_ and **ū**_1_… **ū**_20_, each one accordingly made of l* = h* = 14,400 data points. The *l*_2_-norms of these vectors were finally computed to assemble vector **d**, featuring 40 distance values for each damage case, among which, the first 20 entries are part of the vector **d_u_** and the remaining 20 ones are instead associated with the vector **d_c_**, see Equation (9).

**Figure 4:**
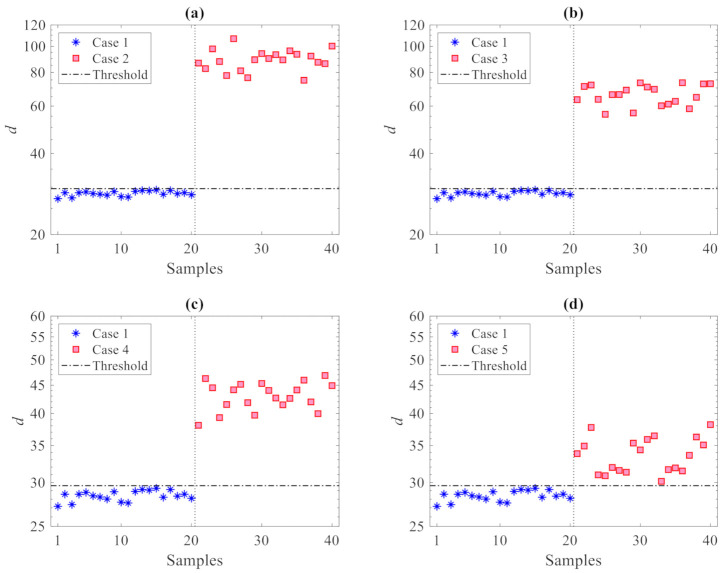
ASCE problem: damage detection via the proposed CMDS method using *p* = 20 partitions: damage (**a**) Case 2, (**b**) Case 3, (**c**) Case 4, and (**d**) Case 5.

The results of the damage detection procedure are displayed in Figure 4, where all the damaged states reported in Table 2 are compared with the undamaged one; in these graphs and similar ones to follow, the horizontal lines refer to the threshold limit computed via the 95% confidence interval of the entries in **d_u_**, see Equation (10), resulting in $\tau_{95\%}=$ 9.6. Irrespective of the damage case, plots show that there are clear deviations in the distance values gathered by **d_c_** above the threshold, therefore, indicative of damage occurrence; the other way around, the values in **d_u_** all fall below the threshold limit, to represent the undamaged state from which $\tau_{95\%}$ has been computed. Such results clearly prove the capability of the proposed method to accurately distinguish between undamaged and damaged states, and thereby detect damage, while also addressing the usual limitations induced by handling high-dimensional feature samples. In the present case, as all the residuals of the AR models at the 12 sensor locations are adopted, damage detection is carried out via 40 distance samples only, in place of the 24,000 original ones in matrices **X** and **Z**.

**Figure 5:**
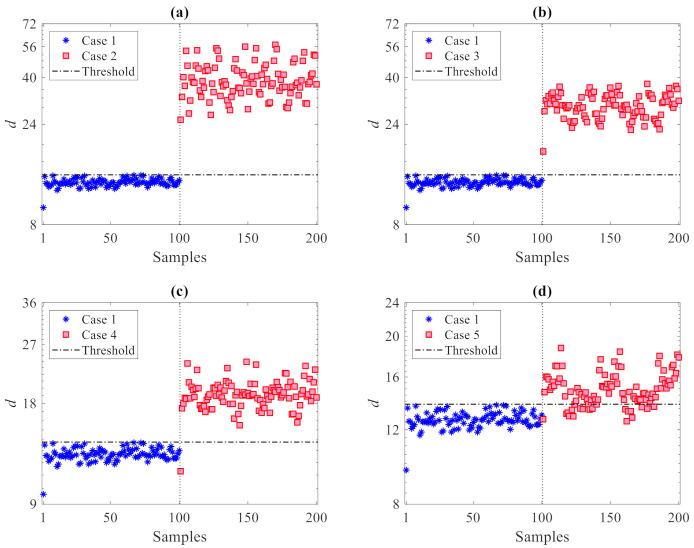
ASCE problem: damage detection via the proposed CMDS method using *p* = 100 partitions: damage (**a**) Case 2, (**b**) Case 3, (**c**) Case 4, and (**d**) Case 5.

To assess the effects of the number *p* of partitions on the damage detection results, Figure 5 provides the results in the case of *p* = 100 partitions used: it can be observed that, again, no values relevant to the first 100 samples (undamaged state) exceed the threshold limit. The distance values relevant to **d_c_** are instead all larger than the threshold in plots 5(a) and 5(b) for the damage in Cases 2 and 3, while some false detections can be observed in plots 5(c) and 5(d) for the damage in Cases 4 and 5. It must be noted that no (Type I) false alarms were encountered; for no partitioning was the undamaged state mistakenly classified as damaged. Additionally, no (Type II) false detections, namely damage states falsely classified by the method as undamaged, were reported for Cases 2 or 3 with any value of *p*. On the one hand, it can be thus emphasized that the use of only a few partitions is also preferable in order to reduce classification errors; on the other hand, Type I errors in all the cases turned out to be zero, testifying the great capability of the proposed method to provide no false alarms.

**Figure 6:**
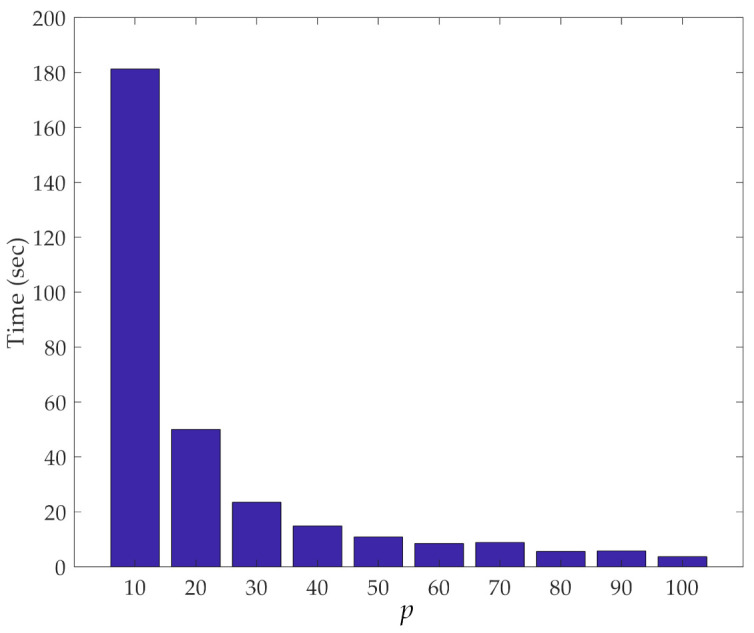
ASCE problem: effect of the number *p* of partitions on the computing time relevant to the iterative loops of the proposed method.

The other important aspect of our dimensionality reduction procedure is its efficiency in terms of computing time. Figure 6 shows this computing time related to the iterative loops of the procedure, at varying values of *p*. Results reported here were obtained with a computer featuring an Intel Core i5-5200@2.20 GHz CPU and 8 GB RAM. It can be observed that the smaller the value of *p*, the longer the time to run over the loops due to the higher number of samples to handle, and for which, pairwise distances must be computed. It is worth mentioning that, despite the rather limited performance of the computer used to run the analyses, the computing time was always constrained to a few seconds; only for *p* = 10 did the entire procedure last around 3 min. Hence, even if the current procedure is proposed in an online fashion, it can be considered performative enough to be re-implemented in the future within an online damage detection approach.

**Figure 7:**
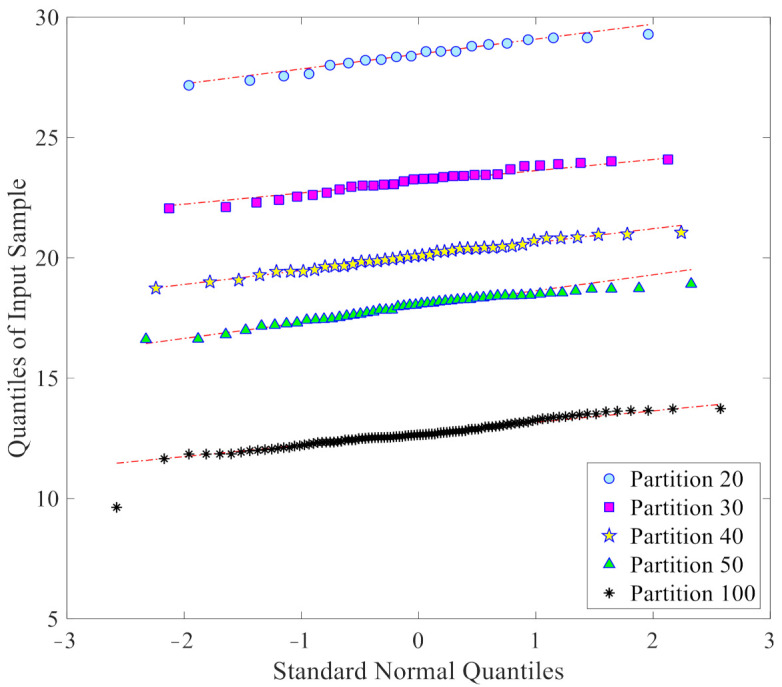
ASCE problem: normality assessment through Q-Q plots of the *l*_2_-norm values in **d_u_**, at varying number *p* of the partitions.

As already mentioned, one of the further advantages of the proposed data-driven approach is to provide samples with a normal or nearly normal distribution, which is best suited for the threshold limit determination via the confidence interval. Figure 7 collects the Q-Q plots of the *l*_2_-norm values in **d_u_**, that are used for threshold determination, again at a varying number of partitions: it clearly emerges that all the sets of the *l*_2_-norms have distributions rather close to the normal one. This capability thus clearly demonstrates the reliability of the obtained threshold limits for damage detection.

**Figure 8:**
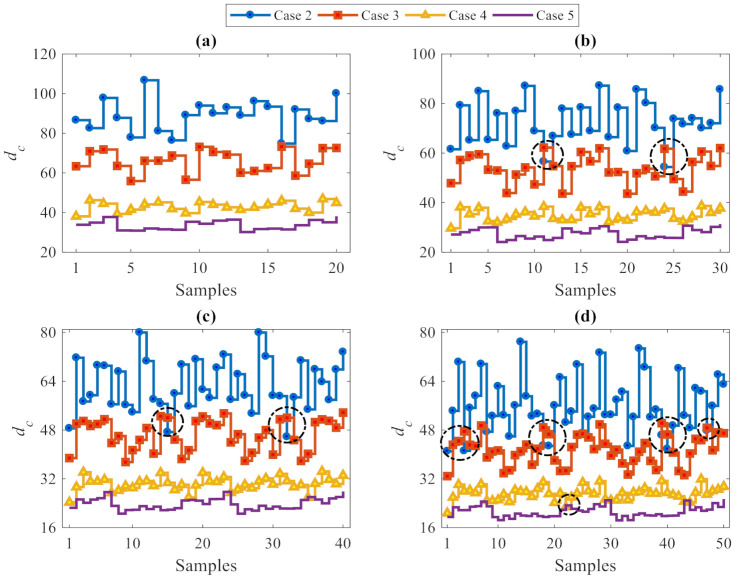
ASCE problem: estimation of damage severity for Cases 2–5, see Table 2, at varying number of partitions (**a**) *p =* 20, (**b**) *p =* 30, (**c**) *p =* 40, and (**d**) *p =* 50.

Besides damage detection, the performance of the proposed data-driven method in estimating the level of damage severity was investigated, using—once more—a varying number of partitions. Figure 8 focuses on the *p* samples of the vector **d_c_** relevant to Cases 2–5. In the charts, the dashed circles highlight (minor) errors in estimating the level of damage severity. More precisely, the *l*_2_-norm values concerning the damage Case 2 are almost always larger than the corresponding values relevant to the other cases; this means that Case 2 features the highest level of damage severity. According to the description of the damaged states in Table 2, this looks reasonable because Case 2 was characterized by the elimination of more bracing systems than damage Cases 3–5. In contrast, the *l*_2_-norm values associated with Case 5 are the smallest and therefore point to the lowest level of damage severity.

It can thus be concluded, that the *l*_2_-norm values increase by increasing the level of damage severity, from Case 5 to Case 2. Therefore, the proposed data-driven method based on the CMDS algorithm was not only able to detect damage accurately, but was also capable of estimating the level of damage severity properly. It was also shown that some erroneous estimates of the damage level have been obtained for analyses featuring *p =* 30, 40 and 50: accordingly, as the best performance was obtained with *p* ≤ 20, a few partitions only should be used if possible. Furthermore, it must be kept in mind that, although the use of few partitions reduces the error rates, it also provides a small, or even too small set of damage indices to be handled at the decision-making stage, and therefore, may not provide adequate outputs. In spite of the similarity in the error rates relevant to solutions linked to *p =* 10 and *p =* 20, the latter solution furnishes more damage indices than the former and, thus, enables a more robust decision to be made about damage occurrence. Therefore, a tradeoff between conflicting outcomes in terms of classification errors and output adequacy represents the criterion to adopt for setting the number of partitions in the proposed CMDS-based method.

### 4.2. Z24 Bridge

**Figure 9:**
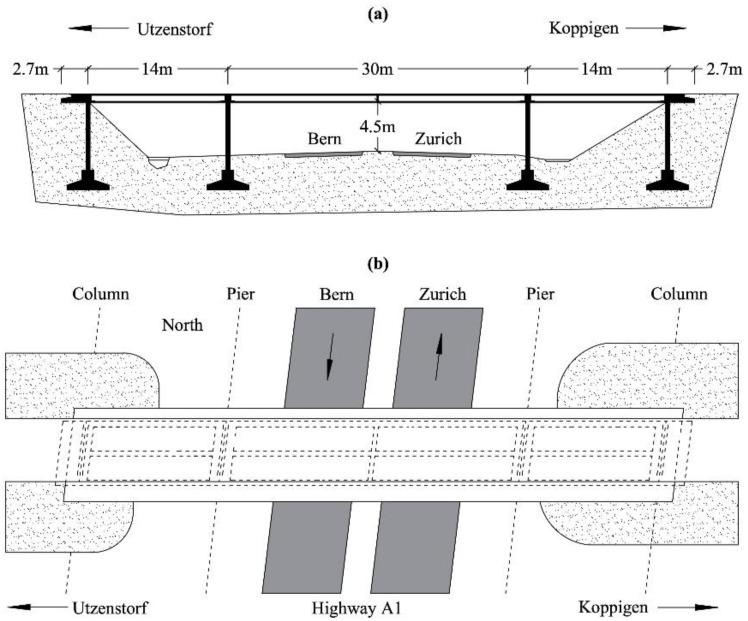
Z24 Bridge: (**a**) longitudinal section and (**b**) top view, adapted from [42].

The Z24 Bridge is a well-known benchmark for long-term SHM [42]. The structure was a post-tensioned box-girder concrete bridge, composed of a main span of 30 m and two side-spans of 14 m, as shown in Figure 9. The bridge was demolished in 1998 to build a new one with a larger side span; before being demolished, it was instrumented and, through a long-term continuous monitoring program, the effects of the environmental variability on damage detection were assessed. Every hour, environmental effects in terms of temperature, wind characteristics, humidity, etc. were measured at several locations with an array of sensors. Acceleration time histories were also acquired with 16 accelerometers located along the bridge with different spatial orientations. Progressive damage tests, including settlement, concrete spalling, landslides, concrete hinge failure, anchor head failure, and the rupture of tendons were carried out to mimic realistic damage scenarios in a controlled way, with the monitoring system always running.

**Figure 10:**
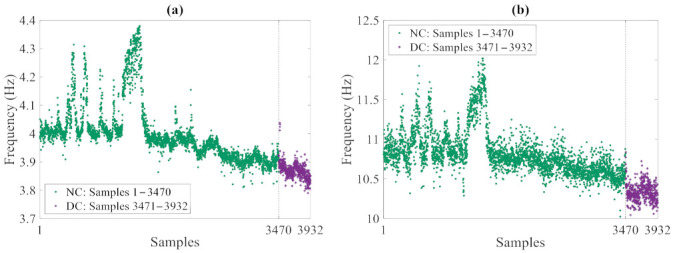
Z24 Bridge: time histories of the monitored vibration frequencies (NC: Normal Condition, DC: Damaged Condition), relevant to the (**a**) first and (**b**) fourth modes.

Starting from the raw data, a modal analysis based on the frequency domain decomposition (FDD) technique was carried out to extract the frequencies of the four fundamental vibration modes and assess their variations due to the environmental conditions. The resulting set of data consists of 3932 measurements, out of which the first 3470 refer to the undamaged, normal condition, and the last 462 ones are associated with the damaged state. Figure 10 collects the exemplary time histories of the modal frequencies relevant to the first and fourth modes: in the plots, the oscillations in the values related to the normal condition were induced by temperature fluctuations in cold periods; these results have clearly testified the high sensitivity of the natural frequencies to the environmental variability. A data normalization procedure based on an auto-associative artificial neural network (AANN) [43] was then adopted to remove such environmental variability from the variation in time of the vibration frequencies and obtain feature samples linked to the damage state only. In compliance with standard approaches in machine learning, 90% of the frequency data regarding the normal condition were handled as the training set, thus including 3123 samples of the four variables (that are the modal frequencies). The last remaining 10% of the frequency values regarding the normal condition, as well as all the values linked to the damaged state were instead handled as the testing set, consisting of 809 samples of the four variables.

The adopted feed-forward AANN architecture consists of three hidden layers representing the mapping, bottleneck, and de-mapping stages, with network outputs that aim at reproducing the corresponding inputs. In the mapping layer, a nonlinear transfer function (namely a sigmoidal one) was used at the neuron level to map the input data onto the bottleneck layer. While the bottleneck layer plays an important role in the functionality of multilayer feedforward networks, as it enforces an internal encoding and a compression of the input data, the relevant type of transfer function does not greatly affect the generality of the network. In the de-mapping layer, again, a non-linear transfer function has been used to decode or de-map the bottleneck compressed data and extract the output data. As the aim of the AANN is to reconstruct the input data, which emerge at the output layer featuring the same size of the input, it provides a filtered version of them. The AANN, thus, represents a smart algorithm for filtering out noise, outliers, and any type of variations in the data due to environmental and/or operational variability [43]. As far as the network hyperparameters are concerned, the number of neurons in each hidden layer was set according to the approach described in [44] and based on the final prediction error. It has turned out that the said number of neurons for the mapping, bottleneck and de-mapping layers has to be, respectively, set to 22, 3, and 22. Finally, as far as the training of the AANN is concerned, the Levenberg–Marquardt back-propagation algorithm was adopted.

To attain data normalization, the AANN was trained to learn the correlations among the features in the training dataset. Once the network was trained to filter out the environmental variability, the residuals between coupled input and output sets were handled as damage-sensitive features for the normal condition. The feature matrix **X** for the undamaged state was, thus, built and consisted of *n* = 3123 residual samples, each one made of the aforementioned *r* = 4 variables. The same procedure was adopted to extract the residuals for the testing set; in this case, the AANN already trained for the normal condition was used to manage the feature set **Z**, consisting of *m* = 809 samples of the same *r* = 4 variables, where *q* = *r* = 4 was again automatically set according to the discussion provided in Section 2.

The proposed data-driven method was then adopted to detect damage. Based on the conclusions drawn for the previous case study regarding the effects of the number of partitions, feature matrices **X** and **Z** were subdivided into *p* = 20, 30, 50, 75, 100, 120, 150, and 200 partitions. Next, to next set the optimal partitioning, the iterative loops of the procedure were run at the varying value of *p* and the rates of Type I, Type II and total errors were obtained as reported in Table 6. Accordingly, the best performance is shown by the solution featuring *p* = 20, with just one error in the entire dataset classification. It can be also seen that the error rates increase by increasing the number of partitions. As already pointed out, smaller values of *p* lead to fewer damage indices to deal with at the decision-making stage, and therefore, reduce the error rates. This outcome turns out to be linked to the handling of smaller sets of damage indices, leading to an easier decision-making process by interpreting and comparing the outputs with each other and with respect to the threshold, distinguishing damaged from undamaged states. For this reason and within certain limits, one can conclude that the use of a small number of partitions provides more reliable results. Though not shown, it must be mentioned that Type I and total error percentages obtained with *p* = 10 turned out to be larger than those reported for *p* = 20, as the relevant damage index dataset does not prove adequate for decision-making. Hence, for this specific case study relevant to the Z24 Bridge, *p* = 20 was targeted as the best choice for damage detection.

**Figure 11:**
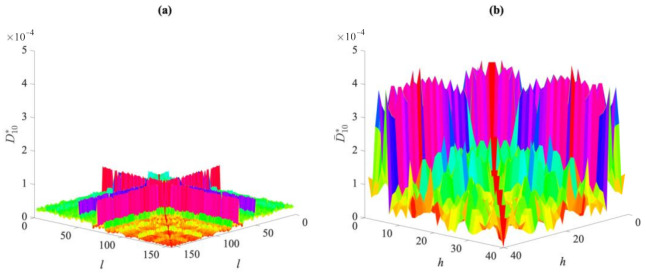
Z24 Bridge, *p* = 20: comparison between the distance values in matrices (**a**) $D_{10}^{*}$ for the normal condition, and (**b**) ${\bar{D}}_{1}^{*}$ for the damaged condition

For the case *p* = 20, Figure 11 compares the values of entries of the distance matrices of the 10th partition (namely of $D_{10}^{*}$ and ${\bar{D}}_{10}^{*}$), regarding the normal and damaged conditions. It can be seen that there is a clear variation between the values corresponding to the two conditions. The proposed methodology was then adopted to ease and speed up the comparison of the two states. After having obtained the distance matrices for all the partitions, the coordinate matrices **U**_1_…**U***_p_* and **Ū**_1_…**Ū***_p_* and their vector forms **u**_1_…**u***_p_* and **ū**_1_… **ū***_p_* were computed, to finally obtain the *l*_2_-norm values and assemble the vector **d** associated with each partition.

**Figure 12:**
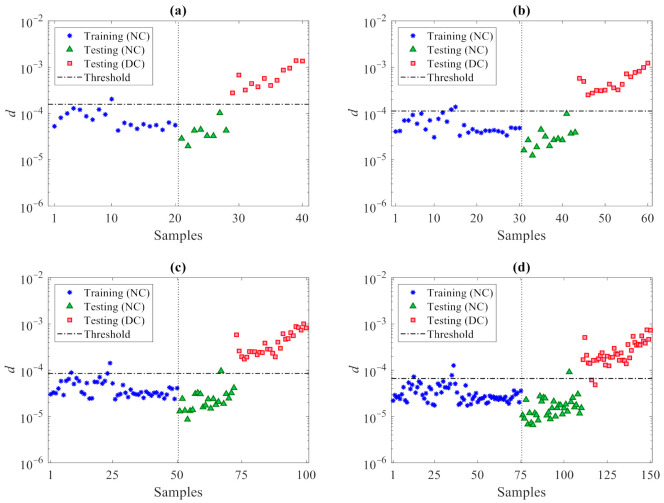

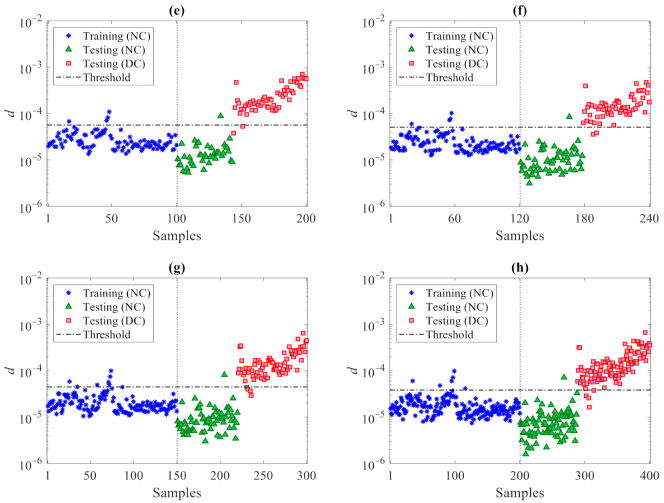
Z24 Bridge: damage detection via the proposed CMDS method at varying number of partitions (**a**) *p* = 20, (**b**) *p* = 30, (**c**) *p* = 50, (**d**) *p* = 75, (**e**) *p* = 100, (**f**) *p* = 120, (**g**) *p* = 150, (**h**) *p* = 200.

The results for varying the number of partitions are shown in Figure 12, where the horizontal lines refer, again, to the threshold limits $\tau_{95\%}$ related to the confidence interval of the entries in **d_u_**. It can be seen that the majority of the training samples for the normal condition in **d** (marked by the blue stars) fall below the threshold limits. Additionally, the values associated with the validation data, namely the testing samples for the normal condition (marked in the charts by the green triangles) do not exceed the thresholds and behave in a way similar to the training data. Conversely, most of the values related to the damaged state—namely, the testing data for the damaged condition (marked by the red squares)—are larger than the threshold limits. This outcome clearly, again, proves the great capability of the proposed data-driven method, ruled by the partitioning strategy and by the CMDS algorithm, to provide an accurate damage detection and, thus, robustly distinguish the damaged state of the structure from the normal condition, even under a strong environmental variability.

**Figure 13:**
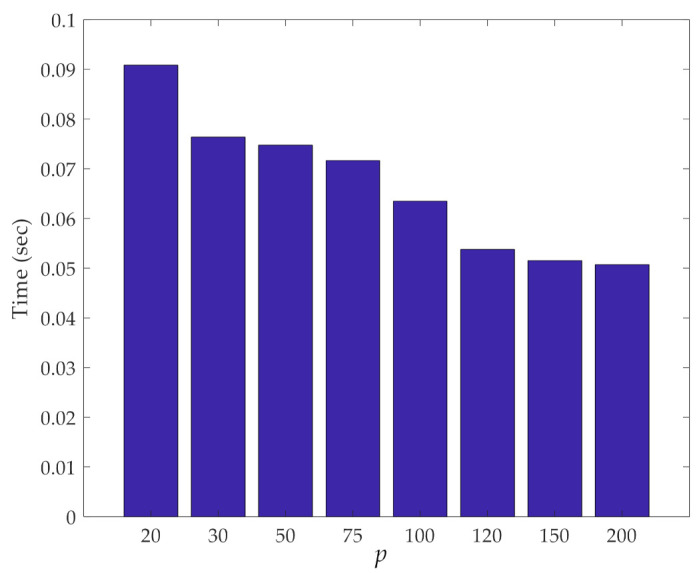
Z24 Bridge: effect of the number *p* of partitions on the computing time relevant to the iterative loops.

The computing time required to perform the iterative loops of the method is shown against the number of partitions in Figure 13. As discussed with reference to the previous case, the cost of the procedure decreases by increasing the number of partitions, due to the reduction in the number of samples in each partition used for the pairwise distance calculation. Even more than in the other case, independently of *p*, the cost is shown to be extremely limited, if not negligible.

**Figure 14:**
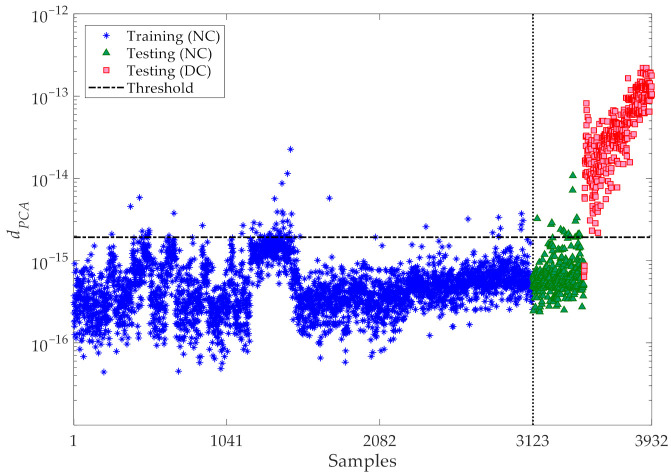
Z24 Bridge: damage detection via the classical PCA method.

Finally, a comparison is reported between the results of the proposed CMDS-based method and those of the PCA technique [45], to prove the superior performance of the former. As discussed in Section 2, PCA is a parametric approach and the number of principle components to be retained in the analysis must be set. This number was determined with the aim to attain 90% of the variance in the training data; accordingly, the principal components retained in the analysis are linked to the eigenvectors, whose eigenvalues overall allow one to attain the mentioned critical threshold of 90% of the variance [46]. The corresponding results of damage detection are depicted in Figure 14. This outcome has been arrived at by handling the same normalized features exploited by the CMDS-based method, after normalization via the AANN, and used as training and testing data samples. Moreover, the outputs of the PCA-based method are based on the Euclidean norms of the residuals between the original normalized features and the reconstructed features obtained via the PCA [45]. It can be observed that a rather large number of outputs (termed *d_PCA_* in the figure) regarding the normal condition exceed the threshold, thereby leading to false alarm or Type I errors. Conversely, some outputs associated with the damaged state fall below the threshold limit, leading—in those cases—to false detection or Type II errors. The comparison between the results relevant to damage detection and those collected in Figure 12 and Figure 14, proves that the proposed CMDS-based method is superior to the PCA-based one, not only because it does not require procedure to set any hyperparameter during the analysis, but also in terms of the smaller error rates obtained.

## 5. Conclusions

In this paper, a data-driven method based on data partitioning and classical multidimensional scaling has been proposed to efficiently and robustly detect damage in structural systems, in the presence of environmental variability. The present study focused on the accuracy and robustness of the method, while also delivering clues to avoid, or reduce to a minimum, false alarms and false damage detection errors. The effectiveness of the proposed method was assessed via two well-known large-scale benchmark examples: the ASCE structure, and the Z24 bridge. The former one has been considered to also show the capability of the approach to classify the severity of damage; while the latter one has been instead adopted to show how the approach, in conjunction with an AANN to preprocess the data, can provide a highly accurate prediction of damage detection by filtering out environmental variability.

Within an unsupervised SHM strategy, for the ASCE structure, each sensor output was fitted with an AR model, which assured accuracy in compliance with the Leybourne–McCabe and the Ljung–Box tests; model residuals were then handled as the high-dimensional damage-sensitive features. For the Z24 bridge, the long-term monitored modal frequencies were instead adopted as model features under strong environmental variability.

For both the case studies, the matrices gathering the damage-sensitive features relevant to the undamaged state (from data collected in the training stage of the SHM procedure) and to the current states (from data collected instead in the damage detection stage of the SHM procedure) were decomposed into smaller subsets. The CMDS coordinates were then recast as damage detection indices based on the relevant *l*_2_-norms, in a partition-wise fashion. Finally, early damage detection was formulated by assessing the drifts of the *l*_2_-norm values in the monitoring stage, away from the baseline set during training. The issue of optimal data partitioning, to maximize the method performance, has also been addressed. The offered methodology can also be seen as a data compression strategy, since damage has been assessed by solving a problem of the same order of the number of subdivisions of the original multivariate datasets, in place of the original order proportional to the number of the acquired measurements.

The results testify that the proposed methodology is highly successful in detecting damage, and distinguishing damaged states from undamaged ones. It has been further shown that the use of a small number of partitions of the original multivariate datasets seems to provide more reliable results, particularly for the Z24 bridge. Moreover, a comparison between the proposed CMDS-based method and the classical PCA technique has shown that the former is superior, as it can be classified as a non-parametric procedure and as it leads to smaller error rates. The ASCE problem has been instead characterized by the best performance in terms of rates of damage detection errors. For both the analyses, the computational cost of the iterative stages of the proposed method was shown to decrease by increasing the number of partitions.

In future work, an investigation will be reported on the effects of the said number of partitions on the time required to process the datasets and provide the vectors of damage indicators. This investigation will be supported by an analysis of the computational complexity of all the algorithmic stages, in order to also discuss the possible parallel implementation of the entire procedure. A deeper exploitation of the MDS coordinates and of the damage indicators will be also attempted, in order to account for the correlation within and across the datasets and help estimate damage location and amplitude. Moreover, since the proposed CMDS-based SHM method handles the data acquired by the sensor network within an offline strategy, a version for online damage detection will be studied.

## Figures and Tables

**Table 1:** ASCE problem: sensor numbering and locations [19].

Direction	Floor No.			
	1	2	3	4
West	4	7	10	13
Center	5	8	11	14
East	6	9	12	15

**Table 2:** ASCE problem: structural features of the undamaged and damaged conditions.

Case No.	Condition	Description
1	Undamaged	Full braced structural system
2	Damaged	Elimination of braces from the east side at all floors
3	Damaged	Elimination of braces from the south-east corner at all floors
4	Damaged	Elimination of braces from the south-east corner at the first and fourth floors
5	Damaged	Elimination of braces from the south-east corner at the first floor

**Table 3:** ASCE problem: Leybourne–McCabe (LMC) test statistics for all sensors and Cases 1–5 of Table 2.

Sensor No.	Case No.				
	1	2	3	4	5
4	0.0001	0.0004	0.0001	0.0001	0.0001
5	0.0057	0.0024	0.0012	0.0009	0.0012
6	0.0019	0.0002	0.0001	0.0001	0.0026
7	0.0002	0.0008	0.0001	0.0001	0.0002
8	0.0011	0.0015	0.0005	0.0005	0.0003
9	0.0145	0.0011	0.0061	0.0042	0.0402
10	0.0002	0.0003	0.0001	0.0002	0.0002
11	0.0011	0.0003	0.0009	0.0003	0.0003
12	0.0002	0.0001	0.0001	0.0002	0.0003
13	0.0069	0.0025	0.0019	0.0031	0.0198
14	0.0051	0.0023	0.0007	0.0007	0.0041

**Table 4:** ASCE problem: AutoRegressive (AR) orders and relevant *p*-values obtained via the LBQ test for each sensor location in Case 1.

Sensor No.	Order	*p*-Value
4	98	0.1295
5	81	0.1048
6	141	0.0799
7	158	0.0581
8	109	0.2513
9	77	0.3833
10	113	0.2376
11	96	0.1437
12	92	0.2812
13	74	0.0794
14	77	0.1027
15	116	0.1778

**Table 5:** ASCE problem: effect of the number *p* of partitions of the matrices **X** and **Z** on the occurrence and percentage of false damage detections for Cases 4 and 5.

No. *p* of Partitions	Case No.	
	4	5
10	0 (0%)	0 (0%)
20	0 (0%)	0 (0%)
30	0 (0%)	2 (6.67%)
40	0 (0%)	2 (5%)
50	0 (0%)	4 (8%)
60	1 (1.67%)	8 (13.34%)
70	1 (1.42%)	10 (14.28%)
80	1 (1.25%)	18 (22.50%)
90	1 (1.11%)	18 (20%)
100	1 (1%)	21 (21%)

**Table 6:** Z24 Bridge: effect of the number *p* of partitions on the occurrence and percentage of Type I, Type II, and total errors in detecting damage.

No. *p* of Partitions	Type I	Type II	Total
20	1 (3.57%)	0 (0%)	1 (2.5%)
30	2 (4.65%)	0 (0%)	2 (3.34%)
50	4 (5.55%)	0 (0%)	4 (4%)
75	4 (3.63%)	2 (5%)	6 (4%)
100	5 (3.50%)	2 (3.51%)	7 (2.5%)
120	5 (2.81%)	2 (3.22%)	7 (2.92%)
150	8 (3.63%)	4 (5%)	12 (4%)
200	7 (2.43%)	4 (3.57%)	11 (2.75%)
